# Geographic disparities and temporal trends regarding access to cancer treatment: a spatial analysis, Brazil, 2015-2022

**DOI:** 10.1590/S2237-96222026v35e20240725.en

**Published:** 2025-12-01

**Authors:** Eduardo Lima de Sousa, Francisco das Chagas de Araújo Rodrigues, Joao Gabriel de Carvalho Saraiva, Ruana Stephany Macedo Santos, Dessana Gomes Medeiros Zagury, Graziela Eulália de Brito

**Affiliations:** 1 Universidade Federal do Piauí, Teresina, PI, Brazil

**Keywords:** Equity in Access to Health Services, Health Information Systems, Unified Health System, Oncology Service, Hospital, Health Inequality Indicators, Equidad en el Acceso a los Servicios de Salud, Sistemas de Información en Salud, Sistema Único de Salud, Servicio de Oncología en Hospital, Indicadores de Desigualdad en Salud

## Abstract

**Objective:**

To analyze spatial clusters and temporal trends regarding patients commuting to access cancer treatment in Brazil between 2015 and 2022.

**Methods:**

This was a spatial analysis using data from hospital and outpatient information systems (2015-2022). Absolute and relative frequencies of treatments (surgery, chemotherapy, and radiotherapy) and commuting patterns were calculated. Cluster analysis (K-means) categorized distances into three intervals: low (2.1-261.4 km), medium (261.6-762.2 km), and high (764.0-3,865.8 km). Temporal trends were assessed by Prais-Winsten regression, estimating annual percentage change (β) and confidence intervals (95%CI) as a measure of dispersion.

**Results:**

Of the 27,204,159 cancer services provided, 55.2% involved displacement to other municipalities. During the period, 3.6% of patients received surgical treatment, 7.1% received radiotherapy, and 89.3% received chemotherapy. There was a reduction in the distances traveled for hospitalization, from 93.0 km in 2015 to 84.2 km in 2022, with an annual decrease of 0.8% (95%CI -0.9; -0.7). For chemotherapy, the reduction was from 87.8 km to 83.5 km, with a variation of -0.4% per year (95%CI -0.4; -0.3). Distances for radiotherapy remained stable, with a slight variation of -0.3% (95%CI -0.9; 0.2).

**Conclusion:**

The reduction in the distance traveled for hospitalization and chemotherapy contrasts with the stability in radiotherapy. The maintenance of long journeys for radiotherapy highlights disparities in the geographical distribution of these services. Thus, the urgency of decentralizing oncology services and investing in regional infrastructure is underscored to ensure access for the population, particularly those residing in rural and remote areas, and to guarantee equitable access to highly complex treatments.

## Resumen

**Objetivo:**

Analizar los aglomerados espaciales y las tendencias temporales en el desplazamiento de pacientes para recibir tratamiento oncológico en Brasil entre 2015 y 2022.

**Métodos:**

Se trató de un análisis espacial utilizando datos de los sistemas de información hospitalaria y ambulatoria (2015-2022). Se calcularon las frecuencias absolutas y relativas de los tratamientos (cirugía, quimioterapia y radioterapia) y los patrones de desplazamiento entre municipios. El análisis de conglomerados (K-means) clasificó las distancias en bajas, medias y altas (intervalos: 2,1-261,4 km; 261,6-762,2 km; 764,0-3865,8 km). Las tendencias temporales se evaluaron mediante regresión de Prais-Winsten, estimando la variación porcentual anual (β) y los intervalos de confianza (IC95%) como medida de dispersión.

**Resultados:**

De las 27 204 159 consultas oncológicas, el 55,2 % implicaron desplazamientos a otros municipios. En el periodo, el 3,6 % de los pacientes recibieron tratamiento quirúrgico; el 7,1 %, radioterapia; y el 89,3 %, quimioterapia. Se observó una reducción en las distancias recorridas para la hospitalización, de 93,0 km en 2015 a 84,2 km en 2022, con una disminución anual del 0,8 % (IC95 % -0,9; -0,7). En el caso de la quimioterapia, la reducción fue de 87,8 km a 83,5 km, con una variación del -0,4 % anual (IC95 % -0,4; -0,3). Las distancias para la radioterapia se mantuvieron estables, con una ligera variación del -0,3 % (IC95 % -0,9; 0,2).

**Conclusión:**

La reducción en la distancia recorrida para hospitalización y quimioterapia contrasta con la estabilidad en radioterapia. El mantenimiento de desplazamientos largos para recibir radioterapia pone de manifiesto las disparidades en la distribución geográfica de estos servicios. Por lo tanto, se destaca la urgencia y se sugiere la descentralización de los servicios oncológicos y la inversión en infraestructura, de forma regionalizada, que garantice el acceso de la población, especialmente de aquellos que residen en zonas rurales y remotas, para garantizar el acceso equitativo a tratamientos de alta complejidad.

**Ethical aspects**

The study used anonymous and publicly available secondary data, so there was no need to submit the project to a research ethics committee.

## Introduction 

The Brazilian National Health System (SUS), as established by Law No. 8,080/1990, is based on organizational principles of decentralization, regionalization, and hierarchization of health services. These principles aim to ensure universal, equitable, and comprehensive access to health care, respecting the regional diversity of the Brazilian territory (1). 

In the context of cancer care, the Brazilian National Policy for Cancer Prevention and Control instituted the Health Care Network for People with Chronic Diseases, organized in a regionalized and hierarchical manner, which considers the need to optimize specialized and high-tech resources. This organization facilitates the strategic distribution of high-complexity oncology care units and centers to meet regional demands, striking a balance between the need for economies of scale and guaranteed access to services (2). Despite this legal and organizational framework, there are still significant challenges to effectively implementing these principles nationwide.

In Brazil, disparities in access to cancer treatment are strongly influenced by the distance patients must travel to reach specialized centers (3). This issue is more critical in rural and remote areas (defined as areas with lower population density, a predominance of agricultural activities, and distance from large urban centers), where the lack of proximity to adequate infrastructure delays diagnosis and treatment, compromising clinical outcomes (4,5). 

The concentration of health services in large urban centers has exacerbated these inequalities, with studies indicating that patients residing far from specialized hubs are less likely to receive early diagnosis and effective treatment (6). In addition, inadequate transportation infrastructure and high commuting costs pose significant barriers, especially for low-income patients, further hindering equitable access to cancer treatment (7).

Inequalities in access to cancer treatment are not only a matter of geography but also reflect deep socioeconomic disparities in the country. Patients in less developed regions often face a shortage of medical services and a general lack of health resources, including medicines and advanced medical technology, which are more readily available in developed urban areas (8-10).

Geographical inaccessibility to medical services is directly related to reduced adherence to these services and ultimately leads to adverse clinical outcomes (11). This reality is fundamental in cancer patients, whose therapeutic regimens, typically multimodal, require multiple interventions, such as surgery, radiotherapy, and chemotherapy, which demand frequent access to treatment units.

Geographic barriers can delay access to necessary treatment, leading to suboptimal care or even preventable deaths. Furthermore, an increased commuting trend among this population is associated with more advanced disease at diagnosis, insufficient therapeutic interventions, poor prognosis, and a deterioration in quality of life. (12).

Despite recent government efforts to decentralize health services and improve access to cancer treatment in underserved areas, inequalities persist. Commuting to urban centers remains a significant challenge, adversely affecting treatment adherence and patients’ quality of life (3,13). It indicates that current policies are still insufficient to fully address the complexities of regional and socioeconomic disparities in health.

This study aimed to analyze spatial clusters and temporal trends in patient commuting patterns for cancer treatment in Brazil from 2015 to 2022.

## Methods 

### Design 

This study involved a cluster analysis and time trend analysis using data from the Hospital Information System and the Outpatient Information System, both of which are part of the IT Department of the Unified Health System. Each data set included records of cancer treatment for patients residing in the Federal District and in one of the 5,570 municipalities located in 26 states, covering the five geographic regions (14).

### Setting 

Through cluster analysis, the groups of cancer patients commuting to the hospital for surgical, chemotherapy, and radiotherapy treatment were mapped. The analysis included only cases in which the pair of municipalities was different, that is, those in which the patient had to travel from their municipality of origin to a different municipality. Areas involving long distances traveled and variations in these distances over the analysis period were identified. 

Data were collected from 2015 to 2022 to examine trends in the distance traveled to access cancer services over time. For each group created in the cluster analysis, the trend over the period was analyzed, as this would enable the definition of variation in distance traveled over time between different clusters with similar characteristics.

### Participants 

The study participants were cancer patients who commuted to another municipality to receive surgical, chemotherapy, or radiotherapy treatment. Only cases involving commuting from one municipality to another were included in the analysis, thus enabling the mapping of patient flows and the identification of commuting patterns.

### Variables 

The variables of interest included the distance traveled by patients to access cancer treatment services, measured in kilometers, and the temporal variation of this distance over the study period. The analysis categorized the data into three groups based on the distance traveled: low (2.1–261.4 km), medium (261.6–762.2 km), and high (764.0–3,865.8 km). These categories enabled a detailed analysis of patients’ behavior in relation to the distances they travel, facilitating a better understanding of their characteristics and potential needs or behavioral patterns.

### Data sources and measurement

Data on the municipality of residence and municipality of treatment for all patients with a primary diagnosis of cancer were extracted from the SUS IT Department database for the period 2015-2022 (15). The codes within the SUS Management System for Procedures, Medications, Orthotics, Prosthetics, and Special Materials selected to represent surgical, chemotherapy, and radiotherapy treatments in this article begin with the following values: Hospital Admission Authorizations for surgical procedures (SUS Hospital Information System code 04.16), High Complexity Outpatient Procedure Authorizations for chemotherapy (SUS Outpatient Information System codes from 03.03.08 to 03.04.02) and radiotherapy (SUS Outpatient Information System code 03.04.01). 

The data set consisted of treatment procedure authorizations (Hospital Admission Authorizations and High Complexity Outpatient Procedure Authorizations), rather than records of individual patients undergoing treatment. Codes C00-C75 from the International Statistical Classification of Diseases and Related Health Problems 10th Revision (ICD-10), corresponding to “Malignant neoplasms, stated or presumed to be primary (of specified sites),” were used to filter patients with a primary diagnosis of cancer (16). These codes included patients of all ages and with different types of cancer.

### Statistical methods

The Distance Matrix Application Programming Interface (API) was used to obtain estimates of road distances between the municipality of residence (origin) and the municipality of treatment (destination) of patients (17). In this context, the analysis included all records in which the pair of municipalities was different. These measurements were based on the best route between the two municipalities, which was derived from the centroids of each municipality.

The clustering technique was employed using the K-means algorithm due to its efficiency and effectiveness in handling large datasets. The K-means algorithm was used to cluster the data, seeking to minimize intra-cluster variation and maximize inter-cluster variation. Initially, the cluster centers were defined randomly, and then each data point was assigned to the nearest center. After each iteration, the centers were recalculated until the model converged (18). 

To determine the appropriate number of clusters, the elbow method was used. This method involved plotting the sum of the quadratic errors within each cluster against the number of clusters. The point at which the curve showed a sharp change indicated the optimal number of clusters. This point was characterized by the least steep decline in the sum of squared errors, suggesting that adding more clusters did not significantly improve the explained variation. The time trend analysis was conducted using the Prais-Winsten generalized linear regression model. In this methodology, the years of the analyzed period (2015-2022) were defined as independent variables (X), while the logarithm to base 10 of the distance traveled per cluster was adopted as the dependent variable (Y). The logarithmic transformation applied to the response variable aimed to minimize heteroscedasticity in the residuals, thereby optimizing the detection of temporal patterns in the regression analysis (18). The model was chosen for its ability to correct for serial autocorrelation, a common phenomenon in time series, where consecutive observations exhibit statistical dependence. 

Serial autocorrelation was assessed using the Durbin-Watson test (19). The annual percentage change (APC) was calculated using the equation: APC=(exp(b)−1)×100, where b represents the slope coefficient of the regression. The 95% confidence interval (95%CI) was defined by applying the same formula to the lower and upper limits of b (denoted by bmin and bmax), resulting in: 95%CI=(exp(bmin)−1)×100 to (exp(bmax)−1)×100. 

The interpretation of trends followed predefined statistical criteria: a decreasing trend (p-value<0.05 with a negative coefficient), an increasing trend (p-value<0.05 with a positive coefficient), and a stable trend (p-value≥0.05), according to a validated methodology (19). 

## Results 

**Figure 1:**
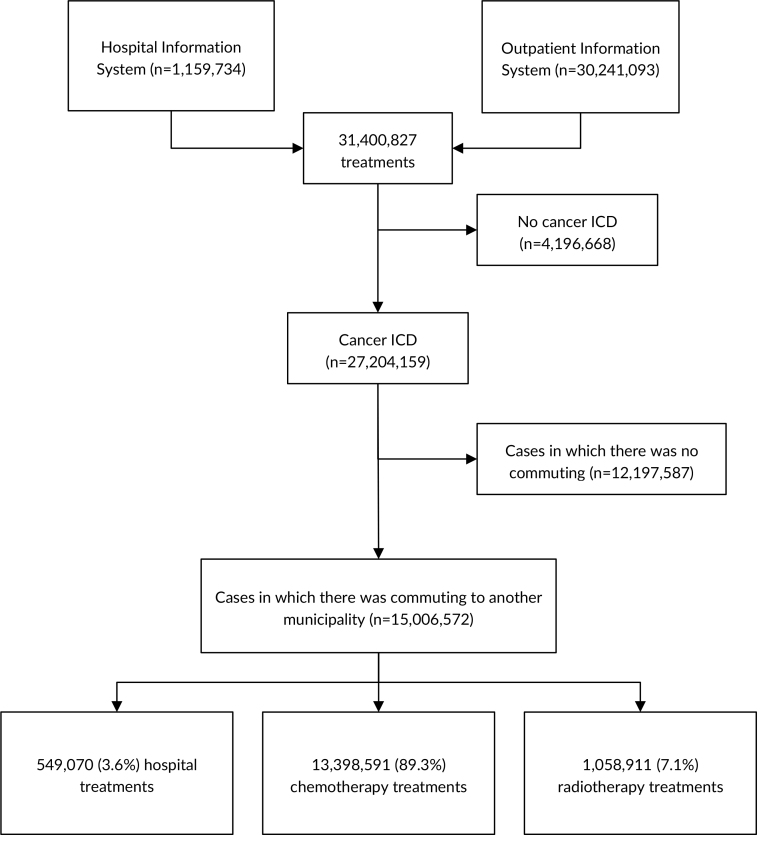
Selection process of individuals who traveled for cancer treatment, based on records from the International Classification of Diseases (ICD) in the Brazilian National Health System. Brazil, 2015-2022 (n=31,400,827)

Between 2015 and 2022, the Hospital Information System and the Outpatient Information System recorded 31,400,827 visits. Of these, 13.4% did not have a cancer ICD code. Among the treatments administered, 44.8% were performed without the need to commute to other municipalities, while 55.2% were transferred to specialized centers. These findings suggest challenges in the distribution and accessibility of cancer care services. For patients who did commute, the distances traveled varied significantly. Those who traveled long distances covered a distance of between 764.0 km and 3,865.8 km; those who traveled medium distances covered a distance of between 261.6 km and 762.2 km; and those who traveled short distances covered a distance of between 2.1 km and 261.4 km. Of the patients who needed to commute between municipalities, 3.6% underwent surgical treatment, 7.1% underwent radiotherapy, and 89.3% underwent chemotherapy (Figure 1).

**Figure 2:**
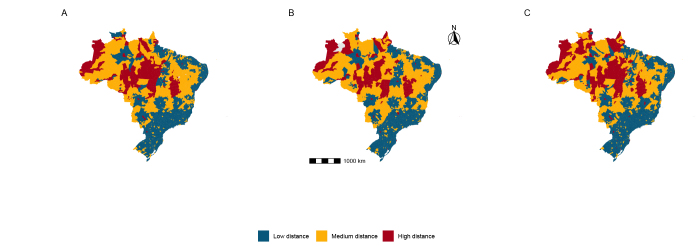
Distances traveled by patients to access cancer treatments in the Brazilian National Health System: (A) hospitalization, (B) radiotherapy, and (C) chemotherapy. Brazil, 2015-2022 (n=15,006,572)

Note: The colors on the maps indicate the categories of distance traveled: blue for short distances (2.1-261.4 km), yellow for medium distances (261.6-762.2 km), and red for long distances (764.0-3,865.8 km).

The maps of distances traveled for cancer treatment in Brazil indicated a concentration of short distances in urban regions for hospitalizations. In rural regions, medium and long distances predominated. Regarding chemotherapy, the distribution revealed that medium distances were extensively traveled, indicating a shortage of nearby treatment centers. Access to radiotherapy was marked by high distances, especially in less accessible areas, thus suggesting significant restrictions in access to these services in remote regions (Figure 2).

**Figure 3:**
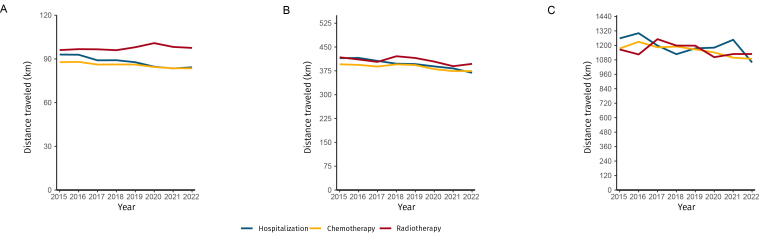
Temporal trend in distances traveled by patients to access cancer treatment in the Brazilian National Health System: (A) short distance; (B) medium distance; and (C) long distance. Brazil, 2015-2022 (n=15,006,572)

Note: The colors on the maps indicate different types of treatment: green for hospitalization, yellow for chemotherapy, and red for radiotherapy.

The distances traveled in kilometers to treatment centers for hospitalization, radiotherapy, and chemotherapy varied according to low, medium, and high distance clusters. For hospitalization, the average distance decreased from 93.0 km in 2015 to 84.2 km in 2022, with an annual coefficient of -0.008 and a percentage reduction of 0.8% (95%CI -0.9; -0.7; p-value<0.001) for patients in low-distance areas. For those at medium distances, the distance decreased from 415.0 km to 369.0 km, representing a 0.7% decrease (95%CI: -0.8; -0.6; p-value<0.001). For those at high distances, there was a reduction from 1,258.1 km to 1,058.2 km, with a variation of -0.7% (95%CI -1.3; -0.1; p-value 0.070), suggesting a more stable trend (Table 1; Figure 3).

**Table 1:** Regression coefficients (β) and 95% confidence intervals (95%CI) for the temporal trend in distances traveled for hospitalization, chemotherapy, and radiotherapy in the Brazilian National Health System. Brazil, 2015-2022 (n=15,006,572)

Variables	Cluster	Coefficient	p-value	Variation (%) (95%CI)	Trend
Hospitalization	Short distance	-0.008	<0.001	-0.8 (-1.0; -0.7)	Decreasing
	Medium distance	-0.007	<0.001	-0.7 (-0.8; -0.7)	Decreasing
	Long distance	-0.007	0.070	-0.7 (-1.3; -0.1)	Stable
Radiotherapy	Short distance	0.002	0.128	0.2 (-0.0; 0.4)	Stable
	Medium distance	-0.003	0.075	-0.3 (-0.6; -0.0)	Stable
	Long distance	-0.003	0.307	-0.3 (-0.9; 0.2)	Stable
Chemotherapy	Short distance	-0.004	<0.001	-0.4 (-0.4; -0.3)	Decreasing
	Medium distance	-0.004	0.012	-0.4 (-0.6; -0.2)	Decreasing
	Long distance	-0.007	0.003	-0.7 (-0.9; -0.4)	Decreasing

The distances traveled for radiotherapy remained stable. In cases of short distances, there was an increase from 96.1 km in 2015 to 97.6 km in 2022, reflecting a percentage increase of 0.2% (95%CI -0.0; 0.4; p-value 0.128). The average distance decreased from 417.6 km to 397.0 km, with a variation of -0.3% (95%CI -0.6; -0.0; p-value 0.075). The highest distance varied from 1,166.2 km to 1,128.6 km, with a change of -0.3% (95%CI -0.9; 0.2; p-value 0.307) (Table 1; Figure 3).

The distances traveled for chemotherapy showed a clear downward trend. For patients traveling short distances, there was a decrease from 87.8 km to 83.5 km, with a percentage reduction of 0.4% (95%CI -0.4; -0.3; p-value<0.001). The average distance decreased from 395.7 km to 374.3 km, with a variation of -0.4% (95%CI -0.6; -0.2; p-value 0.012). For those traveling long distances, there was a decrease from 1,174.4 km to 1,088.4 km, representing a reduction of -0.7% (95%CI -0.9; -0.4; p-value 0.003) (Table 1; Figure 3).

## Discussion 

The results indicated persistent geographic disparities in access to cancer treatment in Brazil, characterized by significant differences in travel patterns. There was heterogeneous progress in access to different types of treatment, with gradual improvement in access to hospitalization and chemotherapy, in contrast to stagnation in access to radiotherapy. This dynamic revealed an unequal decentralization process of oncology services, favoring specific therapeutic modalities to the detriment of others, possibly reflecting differences in implementation costs, infrastructure requirements, and resource allocation decisions.

This study presented significant methodological limitations. The unit of analysis consisted of procedure records (surgery, chemotherapy, and radiotherapy), rather than individual patients, as database linkage techniques that would allow longitudinal patient tracking throughout their treatment were not employed. Thus, the results reflect travel patterns for specific procedures, but do not necessarily capture the full complexity of the cancer care journey, which often involves multiple types of treatment at different times. 

To minimize potential biases related to this limitation, only the first travel record within a 90-day interval was considered for each patient, excluding multiple counts of visits related to the same treatment episode. The use of the municipality centroid as the reference point for calculating distances presented important methodological advantages, such as standardized measurement across all Brazilian municipalities, ease of computational implementation, and comparability between different regions of the country. However, this approach has significant limitations that should be acknowledged. 

The geometric centroid of a municipality is often located in uninhabited areas or regions with low population density, especially in large or irregularly populated municipalities, as is common in rural areas of the Amazon or Brazil’s Central-West. Such a limitation may lead to either underestimation or overestimation of the actual distances traveled by patients, since their actual residence is generally located in population centers that may be far from the geometric centroid. 

Another limitation of this study concerns the analyzed period (2015–2022), which includes the COVID-19 pandemic. During the pandemic, mobility restrictions and the reorganization of health services significantly impacted access to cancer treatment. There was a reduction in cancer diagnosis and treatment, possibly masking the real trend in distance traveled, with likely underreporting of cases and postponement of non-emergency treatments between 2020 and 2022.

The observed reduction in distances for hospitalization and chemotherapy may be related to the expansion of high-complexity oncology care units in previously underserved regions, accompanied by an increase in the distribution of oncology centers. However, significant concentration remains in metropolitan areas (21). Other analyses using an ecological approach in Brazilian territory had already indicated a gradual decentralization of medium-complexity services. In contrast, high-technology services remained concentrated, corroborating the findings regarding stable radiotherapy distances (4,22). 

The prevalence of long distances to access radiotherapy is consistent with international studies (23). In Canada, an inverse relationship was observed between distance and adherence to radiotherapy treatment, with a greater impact on rural populations (24). This geographic barrier contributes to diagnoses at more advanced stages and worse clinical outcomes, making distance a key determinant in the quality of cancer treatment. 

The logistics support system is a crucial element for mitigating the geographic disparities identified (25). However, even in urban contexts, low-income families face barriers to accessing specialized services when relying solely on public transportation, indicating that the mere existence of a service does not guarantee effective access (26). International experience, as documented in Canada, shows that traveling for cancer treatment imposes a significant physical, emotional, and financial burden, often overlooked in health service planning (24). 

The regionalization of oncology services, as advocated by the National Policy for Cancer Prevention and Control, presents a challenge in balancing economies of scale with equitable access. However, concerning access to Primary Health Care services in rural municipalities in Brazil, the importance of hybrid models is highlighted—those that combine selective decentralization with robust referral systems and medical transportation (27). This approach is particularly relevant for radiotherapy, whose high implementation and maintenance costs hinder widespread decentralization, thus requiring complementary strategies such as accommodations for patients and caregivers during treatment.

The impact of the COVID-19 pandemic on access to cancer treatment warrants special attention. International evidence points to a significant reduction in cancer diagnosis and treatment during this period, as seen in the United States, where screenings for breast, colon, prostate, and lung cancers dropped sharply in April 2020 (28). In the Brazilian context, the partial interruption of non-emergency services and intermunicipal mobility restrictions altered the travel patterns identified in this study. It suggests that the modest reductions observed in average distances traveled may not reflect real improvements in access, but rather a decline in the demand for treatment during the health emergency (29). 

Geographic disparities in access to cancer treatment remain a persistent challenge within Brazil’s health system, with limited progress in the decentralization of high-complexity services, especially radiotherapy. The implementation of policies that strengthen regionalization and logistics support systems is crucial to ensure equitable access, particularly for populations living in rural and remote areas. It is imperative to develop strategies that extend beyond the mere geographic distribution of services and address the multiple dimensions of access to healthcare.
