# Supplementary material for: Geographic disparities and temporal trends regarding access to cancer treatment: a spatial analysis, Brazil, 2015-2022
Source: Epidemiol Serv Saude. 2025 Dec 1;35:e20240725. doi: 10.1590/S2237-96222026v35e20240725.en (PMC12668355; doi:10.1590/S2237-96222026v35e20240725.en)
Supplement: Translation

# Translation: Disparidades geográficas e tendência temporal no fluxo de acesso ao tratamento de câncer: análise espacial, Brasil, 2015-2022

**DOI:** 10.1590/S2237-96222026v35e20240725.pt

**Authors:** Eduardo Lima de Sousa, Francisco das Chagas de Araújo Rodrigues, Joao Gabriel de Carvalho Saraiva, Ruana Stephany Macedo Santos, Dessana Gomes Medeiros Zagury, Graziela Eulália de Brito

**Affiliations:** 1 Universidade Federal do Piauí, Teresina, PI, Brasil

**Keywords:** Equidade no Acesso aos Serviços de Saúde, Sistemas de Informação em Saúde, Sistema Único de Saúde, Serviço Hospitalar de Oncologia, Indicadores de Desigualdade em Saúde

## Abstract

**Objetivo:**

Analisar aglomerados espaciais e tendências temporais no deslocamento de pacientes para tratamento oncológico no Brasil entre 2015 e 2022.

**Métodos:**

Tratou-se de análise espacial utilizando dados dos sistemas de informações hospitalares e ambulatoriais (2015-2022). Calcularam-se frequências absolutas e relativas dos tratamentos (cirurgia, quimioterapia e radioterapia) e padrões de deslocamento entre municípios. A análise de aglomerados (K-means) categorizou distâncias em baixa, média e elevada (intervalos: 2,1-261,4 km; 261,6-762,2 km; 764,0-3.865,8 km). Tendências temporais foram avaliadas por regressão de Prais-Winsten, estimando variação percentual anual (β) e intervalos de confiança (IC95%) como medida de dispersão.

**Resultados:**

Dos 27.204.159 atendimentos oncológicos, 55,2% envolveram deslocamento para outros municípios. No período, 3,6% pacientes receberam tratamento cirúrgico; 7,1%, radioterapia; e 89,3%, quimioterapia. Observou-se redução nas distâncias percorridas para hospitalização, de 93,0 km em 2015 para 84,2 km em 2022, com diminuição anual de 0,8% (IC95% -0,9; -0,7). Para quimioterapia, a redução foi de 87,8 km para 83,5 km, com variação de -0,4% ao ano (IC95% -0,4; -0,3). As distâncias para radioterapia permaneceram estáveis, com leve variação de -0,3% (IC95% -0,9; 0,2).

**Conclusão:**

A redução na distância percorrida para hospitalização e quimioterapia contrastam com a estabilidade na radioterapia. A manutenção de deslocamentos extensos para radioterapia evidencia disparidades na distribuição geográfica desses serviços. Assim, destaca-se a urgência e sugere-se a descentralização dos serviços oncológicos e de investimentos em infraestrutura, de forma regionalizada, que assegure o acesso da população, especialmente daquelas que residem em áreas rurais e remotas, para garantir o acesso equitativo a tratamentos de alta complexidade.

**Aspectos éticos**

O estudo utilizou dados secundários anônimos e de acesso público, de forma a não haver a necessidade de submissão do projeto a um comitê de ética em pesquisa.

## Introdução 

O Sistema Único de Saúde (SUS), estabelecido pela Lei nº 8.080/1990, fundamenta-se em princípios organizativos de descentralização, regionalização e hierarquização dos serviços de saúde. Esses princípios visam garantir acesso universal, equitativo e integral à saúde, respeitando as diversidades regionais do território brasileiro (1). 

No contexto da atenção oncológica, a Política Nacional para a Prevenção e Controle do Câncer instituiu a Rede de Atenção à Saúde das Pessoas com Doenças Crônicas, organizada de forma regionalizada e hierarquizada, que considera a necessidade de otimização de recursos especializados e de alta densidade tecnológica. Essa organização prevê a distribuição estratégica de unidades de assistência de alta complexidade em oncologia e centros de assistência de alta complexidade em oncologia para atender às demandas regionais, equilibrando a necessidade de economia de escala com a garantia de acesso aos serviços (2). Apesar desse arcabouço legal e organizativo, persistem desafios significativos na implementação efetiva desses princípios no território nacional.

No Brasil, a disparidade no acesso ao tratamento de câncer é fortemente influenciada pela distância que os pacientes devem percorrer até alcançar centros especializados (3). Essa questão é mais crítica em áreas rurais e remotas (definidas como áreas com menor densidade demográfica, predominância de atividades agrícolas e distantes de grandes centros urbanos), onde a falta de proximidade com infraestruturas adequadas atrasa diagnósticos e tratamentos, o que compromete os resultados clínicos (4,5). 

A centralização dos serviços de saúde em grandes centros urbanos exacerbou essas desigualdades, com estudos indicando que pacientes que residem longe de centros especializados têm menor probabilidade de receber diagnósticos precoces e tratamentos eficazes (6). Além disso, a infraestrutura de transporte inadequada e os altos custos associados ao deslocamento impõem barreiras significativas, especialmente para pacientes de baixa renda, o que dificulta ainda mais o acesso equitativo ao tratamento oncológico (7).

As desigualdades no acesso ao tratamento de câncer não são apenas uma questão de geografia, mas também refletem disparidades socioeconômicas profundas do país. Pacientes em regiões menos desenvolvidas enfrentam a escassez de serviços médicos e a falta geral de recursos de saúde, como medicamentos e tecnologia médica avançada, que são mais acessíveis em áreas urbanas desenvolvidas (8-10).

A inacessibilidade geográfica aos serviços médicos está diretamente relacionada à reduzida adesão a esses serviços e culmina em desfechos clínicos adversos (11). Essa realidade assume particular importância em pacientes oncológicos, cujos regimes terapêuticos, tipicamente multimodais, exigem múltiplas intervenções, como cirurgia, radioterapia e quimioterapia, o que demanda frequente acesso a unidades de tratamento.

Obstáculos geográficos podem retardar o acesso ao tratamento necessário, levando a uma prestação de cuidados subótima ou até fatalidades evitáveis. Além disso, o aumento do deslocamento nessa população está associado à apresentação de casos em estágios mais avançados no momento do diagnóstico, às intervenções terapêuticas insuficientes, aos prognósticos desfavoráveis e à deterioração da qualidade de vida (12).

Apesar dos esforços recentes do governo para descentralizar os serviços de saúde e melhorar o acesso ao tratamento de câncer em áreas subatendidas, as desigualdades persistem. O deslocamento para centros urbanos continua sendo um desafio significativo, que afeta adversamente a adesão ao tratamento e a qualidade de vida dos pacientes (3,13). Isso indica que as políticas atuais ainda são insuficientes para abordar completamente as complexidades das disparidades regionais e socioeconômicas em saúde.

Este estudo teve como objetivo analisar os aglomerados espaciais e as evoluções temporais no deslocamento para tratamento oncológico no Brasil durante o período 2015-2022.

## Métodos 

### Delineamento 

Tratou-se de análise de aglomerados e de tendência temporal que utilizou dados do Sistema de Informações Hospitalares e do Sistema de Informações Ambulatoriais, ambos originados do Departamento de Informática do Sistema Único de Saúde. Cada conjunto de dados incluiu registros de tratamento oncológico de pacientes residentes no Distrito Federal e em um dos 5.570 municípios localizados em 26 estados , contemplando as cinco regiões geográficas (14).

### Contexto 

Por meio da análise de aglomerados, mapearam-se os grupos de deslocamento e o fluxo dos pacientes oncológicos do local de residência até o hospital para tratamento cirúrgico, quimioterápico e radioterápico. A análise incluiu apenas casos em que o par de municípios foi diferente, ou seja, aqueles em que o paciente teve que se deslocar do município de origem para um município diferente. Foram identificadas zonas que demandavam elevada distância percorrida e a variação dessa distância ao longo do período de análise. 

Foram coletados dados de 2015 a 2022 com a finalidade de examinar as tendências na distância percorrida para o acesso aos serviços oncológicos ao longo do tempo. Para cada grupo criado na análise de aglomerados, a tendência ao longo do período foi analisada, pois assim seria possível definir a variação na distância percorrida ao longo do tempo entre diferentes agrupamentos que apresentavam características semelhantes.

### Participantes 

Os participantes do estudo foram pacientes oncológicos que se deslocaram para outro município a fim de receber tratamento cirúrgico, quimioterápico ou radioterápico. Apenas os casos em que houve deslocamento entre municípios distintos foram incluídos na análise, permitindo o mapeamento do fluxo de pacientes e a identificação de padrões de deslocamento.

### Variáveis 

As variáveis de interesse incluíram a distância percorrida pelos pacientes para acessar os serviços de tratamento oncológico, medida em quilômetros, e a variação temporal dessa distância ao longo do período de estudo. A análise categorizou os dados em três grupos com base na distância percorrida: baixa (2,1-261,4 km), média (261,6-762,2 km) e elevada (764,0-3.865,8 km). Essas categorias permitiram a análise detalhada do comportamento dos pacientes em relação às distâncias que percorrem, o que facilitou a compreensão mais profunda de suas características e potenciais necessidades ou padrões de comportamento.

### Fonte de dados e mensuração

Os dados do município de residência e do município de tratamento de todos os pacientes com diagnóstico principal de câncer foram extraídos do banco de dados do Departamento de Informática do SUS, referentes ao período 2015-2022 (15). Os códigos do Sistema de Gerenciamento da Tabela de Procedimentos, Medicamentos e Órteses, Próteses e Materiais Especiais do SUS selecionados para representar os tratamentos cirúrgicos, quimioterápicos e radioterápicos neste artigo iniciam com os seguintes valores: Autorizações de Internação Hospitalar para procedimentos cirúrgicos (código do Sistema de Informações Hospitalares do SUS 04.16), Autorizações de Procedimentos Ambulatoriais de Alta Complexidade para quimioterapia (códigos do Sistema de Informações Ambulatoriais do SUS de 03.03.08 a 03.04.02) e radioterapia (código do Sistema de Informações Ambulatoriais do SUS 03.04.01). 

O conjunto de dados consistiu em autorizações de procedimentos de tratamento (Autorizações de Internação Hospitalar e de Procedimentos Ambulatoriais de Alta Complexidade), e não em registros de pacientes individuais em tratamento. Os códigos C00-C75 da 10ª revisão da Classificação Internacional de Doenças (CID-10), correspondentes a “neoplasias malignas, declaradas ou presumidas como primárias (de sítios especificados)”, foram utilizados para filtrar os pacientes com diagnóstico primário de câncer (16). Esses códigos incluíram pacientes de todas as idades e com diferentes tipos de cânceres.

### Métodos estatísticos

A Interface de Programação de Aplicações (API) Distance Matrix foi utilizada para obter estimativas das distâncias rodoviárias entre o município de residência (procedência) e o município de tratamento (destino) dos pacientes (17). Nesse contexto, a análise incluiu todos os registros em que o par de municípios foi diferente. Essas medidas foram baseadas na melhor rota entre os dois municípios, derivada do centroide de cada município.

A técnica de aglomerados foi empregada utilizando o algoritmo K-means devido à sua eficiência e eficácia em lidar com grandes conjuntos de dados. O algoritmo K-means foi utilizado para agrupar os dados, buscando minimizar a variação intra-cluster e maximizar a variação entre aglomerados. Inicialmente, os centros dos aglomerados foram definidos de forma aleatória e, em seguida, cada ponto de dado foi atribuído ao centro mais próximo. Após cada iteração, os centros foram recalculados até a convergência do modelo (18). 

Para determinar o número adequado de aglomerados, utilizou-se o método do cotovelo. Esse método envolveu a plotagem da soma dos erros quadráticos dentro de cada aglomerado em relação ao número de aglomerados. O ponto em que a curva apresentou uma mudança acentuada indicou o número ideal de aglomerados. Esse ponto foi caracterizado pelo declínio menos acentuado na soma dos erros quadráticos, sugerindo que a adição de mais aglomerados não melhorou de forma significativa a variação explicada. A análise de tendência temporal foi conduzida por meio do modelo de regressão linear generalizada de Prais-Winsten. Nessa metodologia, os anos do período analisado (2015-2022) foram definidos como variáveis independentes (X), enquanto o logaritmo na base 10 da distância percorrida por aglomerado foi adotado como variável dependente (Y). A transformação logarítmica aplicada à variável resposta teve como objetivo minimizar a heterocedasticidade dos resíduos, otimizando a detecção de padrões temporais na análise de regressão (18). O modelo foi escolhido pela capacidade de corrigir autocorrelação serial, comum em séries temporais, em que observações consecutivas apresentam dependência estatística. 

A avaliação da autocorrelação serial foi realizada utilizando o teste de Durbin-Watson (19). A variação percentual anual (APC) foi calculada utilizando a equação: APC=(exp(b)−1)×100, em que b representa o coeficiente angular da regressão. O intervalo de confiança de 95% (IC95%) foi definido aplicando-se a mesma fórmula aos limites inferior e superior de b (denotados por bmin e bmax), resultando em: IC95%=(exp(bmin)−1)×100 a (exp(bmax)−1)×100. 

A interpretação das tendências seguiu critérios estatísticos predefinidos: tendência decrescente (p-valor<0,05 com coeficiente negativo), tendência ascendente (p-valor<0,05 com coeficiente positivo) e tendência estável (p-valor≥0,05), conforme metodologia validada (19).

## Resultados 

Entre 2015 e 2022, o Sistema de Informações Hospitalares e o Sistema de Informações Ambulatoriais registraram 31.400.827 atendimentos. Destes, 13,4% não apresentaram CID de câncer. Dos tratamentos administrados, 44,8% foram realizados sem o deslocamento para outros municípios, enquanto 55,2% foram transferidos para centros especializados, o que sugeriu desafios na distribuição e na acessibilidade dos serviços de saúde oncológica. Para os pacientes que se deslocaram, as distâncias percorridas variaram significativamente. Aqueles que viajaram elevada distância percorreram entre 764,0 km e 3.865,8 km; os de média distância, entre 261,6 km e 762,2 km; e os de baixa distância, de 2,1 km a 261,4 km. Dos pacientes que se deslocaram, 3,6% foram submetidos a tratamento cirúrgico; 7,1%, a tratamento radioterápico; e 89,3%, a quimioterapia (Figura 1).

**Figura 1:** Processo de seleção dos indivíduos que se deslocaram para tratamento oncológico conforme registros da Classificação Internacional de Doenças (CID) no Sistema Único de Saúde. Brasil, 2015-2022 (n=31.400.827)

Os mapas de distância percorrida para tratamentos de câncer no Brasil indicaram a concentração de baixas distâncias em regiões urbanas para hospitalizações. Em regiões rurais, predominaram distâncias médias e elevadas. No contexto da quimioterapia, a distribuição mostrou que médias distâncias foram extensivamente percorridas, o que refletiu a escassez de centros de tratamento mais próximos. O acesso à radioterapia foi marcado por elevadas distâncias, especialmente em áreas menos acessíveis, e sugeriu restrição significativa no acesso a esses serviços em regiões remotas (Figura 2).

**Figura 2:** Distâncias percorridas pelos pacientes para acesso a tratamentos oncológicos no Sistema Único de Saúde: (A) hospitalização, (B) radioterapia e (C) quimioterapia. Brasil, 2015-2022 (n=15.006.572)

Nota: As cores nos mapas indicam as categorias de distância percorrida: azul para baixa distância (2,1-261,4 km), amarelo para média distância (261,6-762,2 km) e vermelho para elevada distância (764,0-3.865,8 km).

As distâncias percorridas em quilômetros até os centros de tratamento para hospitalização, radioterapia e quimioterapia variaram conforme os aglomerados de baixa, média e elevada distâncias. Na hospitalização, a distância média diminuiu de 93,0 km em 2015 para 84,2 km em 2022, com coeficiente anual de -0,008 e redução percentual de 0,8% (IC95% -0,9; -0,7; p-valor<0,001) para os pacientes em áreas de baixa distância. Para os de média distância, a distância reduziu de 415,0 km para 369,0 km, com diminuição de 0,7% (IC95% -0,8; -0,6; p-valor<0,001). Os de elevada distância apresentaram redução de 1.258,1 km para 1.058,2 km, com variação de -0,7% (IC95% -1,3; -0,1; p-valor 0,070), sugerindo tendência mais estável (Tabela 1; Figura 3).

**Tabela 1:** Coeficientes de regressão (β) e intervalos de confiança de 95% (IC95%) para tendência temporal das distâncias percorridas para hospitalização, quimioterapia e radioterapia no Sistema Único de Saúde. Brasil, 2015-2022 (n=15.006.572)

Variáveis	Aglomerado	Coeficiente	p-valor	Variação (%) (IC95%)	Tendência
Hospitalização	Baixa distância	-0,008	<0,001	-0,8 (-1,0; -0,7)	Decrescente
	Média distância	-0,007	<0,001	-0,7 (-0,8; -0,7)	Decrescente
	Elevada distância	-0,007	0,070	-0,7 (-1,3; -0,1)	Estável
Radioterapia	Baixa distância	0,002	0,128	0,2 (-0,0; 0,4)	Estável
	Média distância	-0,003	0,075	-0,3 (-0,6; -0,0)	Estável
	Elevada distância	-0,003	0,307	-0,3 (-0,9; 0,2)	Estável
Quimioterapia	Baixa distância	-0,004	<0,001	-0,4 (-0,4; -0,3)	Decrescente
	Média distância	-0,004	0,012	-0,4 (-0,6; -0,2)	Decrescente
	Elevada distância	-0,007	0,003	-0,7 (-0,9; -0,4)	Decrescente

**Figura 3:** Tendência temporal das distâncias percorridas pelos pacientes para acesso a tratamentos oncológicos no Sistema Único de Saúde: (A) baixa distância; (B) média distância; e (C) elevada distância. Brasil, 2015-2022 (n=15.006.572)

Nota: As cores nos mapas indicam diferentes tipos de tratamento: verde para hospitalização, amarelo para quimioterapia e vermelho para radioterapia.

As distâncias percorridas mostraram-se estáveis para a radioterapia. Nos casos de baixa distância, houve aumento de 96,1 km em 2015 para 97,6 km em 2022, o que refletiu incremento percentual de 0,2% (IC95% -0,0; 0,4; p-valor 0,128). As distâncias médias apresentaram diminuição de 417,6 km para 397,0 km, com variação de -0,3% (IC95% -0,6; -0,0; p-valor 0,075). A elevada distância variou de 1.166,2 km para 1.128,6 km, com alteração de -0,3% (IC95% -0,9; 0,2; p-valor 0,307) (Tabela 1; Figura 3).

As distâncias percorridas evidenciaram clara tendência de redução para quimioterapia. Para os pacientes de baixa distância, houve diminuição de 87,8 km para 83,5 km, com redução percentual de 0,4% (IC95% -0,4; -0,3; p-valor<0,001). A média distância caiu de 395,7 km para 374,3 km, com variação de -0,4% (IC95% -0,6; -0,2; p-valor 0,012). Para os de elevada distância, observou-se diminuição de 1.174,4 km para 1.088,4 km, com redução de -0,7% (IC95% -0,9; -0,4; p-valor 0,003) (Tabela 1; Figura 3).

## Discussão 

Os resultados indicaram persistentes disparidades geográficas no acesso ao tratamento oncológico no Brasil, caracterizadas por diferenças significativas nos padrões de deslocamento. Observou-se evolução heterogênea na acessibilidade aos diferentes tipos de tratamento, com melhoria gradual no acesso à hospitalização e quimioterapia, em contraste com a estagnação no acesso à radioterapia. Essa dinâmica revelou um processo de descentralização desigual dos serviços oncológicos, que favorece determinadas modalidades terapêuticas em detrimento de outras, possivelmente refletindo diferenças nos custos de implementação, requisitos de infraestrutura e decisões de alocação de recursos.

Este estudo apresentou limitações metodológicas significativas. A unidade de análise foi constituída pelos registros de procedimentos (cirurgia, quimioterapia e radioterapia), em vez de pacientes individuais, uma vez que não foram empregadas técnicas de pareamento de banco de dados que permitiriam o acompanhamento longitudinal dos pacientes ao longo de seu tratamento. Desse modo, os resultados refletiram padrões de deslocamento para procedimentos específicos, mas não capturaram necessariamente a complexidade total da jornada do paciente oncológico, que frequentemente requer múltiplos tipos de tratamento em diferentes momentos. 

Para minimizar potenciais vieses relacionados à limitação, considerou-se apenas o primeiro registro de deslocamento de cada paciente em um intervalo de 90 dias, excluindo múltiplas contagens de visitas relacionadas ao mesmo episódio de tratamento. O uso do centroide do município como ponto de referência para o cálculo das distâncias apresentou vantagens metodológicas importantes, como a padronização da medida para todos os municípios brasileiros, a simplicidade na implementação computacional e a comparabilidade entre diferentes regiões do país. No entanto, essa abordagem possui limitações significativas que devem ser reconhecidas. 

O centroide geométrico de um município frequentemente está situado em áreas inabitadas ou com baixa densidade populacional, especialmente em municípios de grande extensão territorial ou com distribuição populacional irregular, como é comum em áreas rurais da Amazônia ou do Centro-Oeste brasileiro. Essa limitação pode levar tanto à subestimação quanto à superestimação das distâncias realmente percorridas pelos pacientes, pois a residência efetiva dos pacientes geralmente se concentra em núcleos populacionais que podem estar distantes do centroide geométrico. 

Outra limitação deste estudo esteve relacionada ao período analisado (2015-2022), que incluiu a pandemia de covid-19. Durante a pandemia, as restrições de mobilidade e a reorganização dos serviços de saúde impactaram significativamente o acesso ao tratamento oncológico. Houve redução no diagnóstico e tratamento de câncer, possivelmente mascarando a tendência real de evolução nas distâncias percorridas, com provável subnotificação de casos e adiamento de tratamentos não emergenciais entre 2020 e 2022.

A redução das distâncias para hospitalização e quimioterapia pode estar relacionada à expansão das unidades de assistência de alta complexidade em oncologia para regiões anteriormente não cobertas, com aumento na distribuição de centros oncológicos, embora ainda persista concentração significativa em áreas metropolitanas (21). Outras análises, utilizando metodologia ecológica no território brasileiro, já indicavam descentralização gradual dos serviços de média complexidade, enquanto os serviços de alta tecnologia permaneciam concentrados, corroborando os achados quanto à estabilidade da distância para radioterapia (4,22). 

A prevalência de grandes distâncias para acesso à radioterapia está em consonância com estudos internacionais (23). No Canadá, foi observada relação inversamente proporcional entre distância e adesão ao tratamento radioterápico, com impacto mais acentuado em populações de áreas rurais (24). Essa barreira geográfica contribui para diagnósticos em estágios mais avançados e piores desfechos clínicos, trazendo a distância como fator determinante na qualidade do tratamento oncológico. 

O sistema de apoio logístico é um elemento crucial para mitigar as disparidades geográficas identificadas (25). No entanto, mesmo em contextos urbanos, famílias de baixa renda enfrentam barreiras para acessar serviços especializados quando dependem exclusivamente do transporte público, o que indica que a simples existência do serviço não garante acesso efetivo (26). A experiência internacional, documentada no Canadá, evidencia que o deslocamento para tratamento oncológico impõe sobrecarga física, emocional e financeira significativa, frequentemente negligenciada no planejamento de serviços de saúde (24). 

A regionalização dos serviços oncológicos, conforme preconizado pela Política Nacional para a Prevenção e Controle do Câncer, enfrenta o desafio de equilibrar economia de escala com equidade no acesso. No entanto, com relação à acessibilidade aos serviços de Atenção Primária à Saúde em municípios rurais do Brasil, destaca-se a importância de modelos híbridos que combinem descentralização seletiva com robustos sistemas de referência e transporte sanitário (27). Tal abordagem mostra-se particularmente relevante para a radioterapia, cujos elevados custos de implementação e manutenção dificultam sua ampla descentralização, o que demanda estratégias complementares como alojamentos para pacientes e acompanhantes durante o período de tratamento.

O impacto da pandemia de covid-19 no acesso ao tratamento oncológico requer atenção especial. Evidências internacionais apontam para redução significativa no diagnóstico e tratamento de câncer durante esse período, a exemplo dos Estados Unidos, onde os exames para câncer de mama, cólon, próstata e pulmão caíram drasticamente em abril de 2020 (28). No contexto brasileiro, a interrupção parcial de serviços não emergenciais e as restrições de mobilidade intermunicipais alteraram os padrões de deslocamento identificados neste estudo. Isso sugere que as modestas reduções observadas na distância média percorrida podem não refletir melhoria real no acesso, mas sim redução na busca por tratamento durante a emergência sanitária (29). 

As disparidades geográficas no acesso ao tratamento oncológico constituem desafio persistente no sistema de saúde brasileiro, com avanços limitados na descentralização de serviços de alta complexidade, especialmente radioterapia. A implementação de políticas que fortaleçam a regionalização e os sistemas de apoio logístico é crucial para garantir acesso equitativo, particularmente para populações residentes em áreas rurais e remotas. Torna-se imperativo o desenvolvimento de estratégias que transcendam a mera distribuição geográfica de serviços e abordem as múltiplas dimensões do acesso à saúde.
